# Editorial

**Published:** 1858-06

**Authors:** 


### Editorial.

### THE AMERICAN JOURNAL ON EROSION.

We (ourself) have been an humble instrument in the hands of Providence in greatly extending the editorial department of the "American Journal." It is true the "senior editor" apologizes for noticing us; for though he considers us an "anonymous friend," yet it is seldom he notices "the strictures of anonymous writers." We by no means intended to be considered "anonymous," and would here remark, that when any thing in the editorial or review department of the Register appears over the initial of either of the editors, the one whose name corresponds with the initial may be held responsible. We can not, like our friends of the "American Journal," speak of the "senior editor" and the "junior;" for by birth, profession and editorial labor, we are equal in age. The W. appended to the article spoken of by the "senior editor,!' therefore, stands for George Watt, as all save the American Journal, no doubt, knew at a single glance. The Journal once thought, if we mistake not, that an editor's initials were almost sufficient acknowledgement to another periodical fo a borrowed article; but now, in interpreting such characters, it falls behind the Irishman who mistook a cartridge box for a bible, but soon corrected his mistake by discovering the initials G., for George, and R., for God bless him.

The expression with which we took issue is as follows:—"Again, they oftentimes meet with caries, or erosion, if they pleased to call it such, *immediately after* the eruption of the teeth from the gums, *showing* that it was to the action of the fluid contained in the sacks of the teeth—that decomposition was attributable." The italics are ours. We insisted that the discovery of erosion *after* the teeth are through the gums does not *show* that it existed *before* the tooth was through the sac, and so we insist still. In taking this position, however, we were fully aware that we differed with the old French authors to whom the "senior editor" refers, to prove that his position is an old one; and we were aware that each of them held "a deservedly high rank," but that did not frighten us; for, in dissenting from the views of the "senior editor," we differed with one who holds *a deservedly higher rank than any of them.* And the age of a doctrine signifies nothing. To fall into an error because it is an hundred years old, is no more excusable than to fall into a pit because it is a hundred feet deep.

But the "senior editor" tells us, "We have two jaws in each of which the crowns of twelve of the permanent teeth, in a dentinified and enameled condition, are still inclosed in their alveolar cells, and nearly all of these are marked by erosion." Now, if our remarks brought out this statement, we are amply repaid for our trouble in writing the review. We inquired, "Did Professor H. ever find this erosion *before* the eruption of the teeth from the gums?" and he gives us a case exactly in point. This is more satisfactory than all the *inferences* ever deduced from the condition of the teeth after eruption, by himself or his French authorities. We hope to have the pleasure of seeing these same jaws; and if the "senior editor" will favor us with the particulars of the case, he will confer a special favor on us western barbarians. But, in view of his arduous labors, we would be sorry to urge even this, however gratifying it would be.

We are charged with misquotation, in noticing the "senior editor's" remarks on the removal of the roots of the temporary teeth. We must plead, "Not Guilty." The official report was furnished by the "News Letter," and it says "the extraneous substance," not *carneous;* and as the report was received, read, printed, and almost memorized by us before the Journal arrived, we never looked at its copy of the report; but, if the word *carneous* is substituted for "extraneous," then the Journal, and not the Register, has misquoted.

We inquired what acid is exhaled by the "extraneous" or *carneous* substance; and we did so simply because we desired to knew. We had no disposition to dispute the principle stated. If not convenient for the "senior" will the junior editor *magnify his office* by enlightening us? And we wonld like to know if the "senior editor" wishes to be understood that, in erosion, or caries, the acting acid usually displaces the phosphoric, and takes the lime. W.

---

### "CHEMISTRY AMONG DENTISTS."

In the April number of the "American Journal" we have the interesting and startling announcement that one of the editors, the junior probably, *believes in chemistry.* And, from what follows, some might fancy that he scarcely allows anybody else to believe in it, at least, unless he holds it according to the promulgations of the *big book.* "It is our own department," he says, with mild symptoms of an intimation that he don't wish it to be considered anybody else's. Now, from observing the motions of the heavenly bodies, we were convinced some time ago that the sun rises in Baltimore; and we have suspected that the old moons are returned thither to be worked up into stars, yet we have still tried to journey on in the paths of science, by the aid of such lamp-light as we are permitted to enjoy.

Ever since the discovery of the fact that the decay of the teeth is caused by chemical action, the *chemistry of caries* has been the important question in dental science. Now, when a few years ago, we learned that a "Dental Chemistry and Metallurgy" was about to be published, we hailed the announcement with delight, and, rather than wait for its regular issue, we paid the publishers for advance sheets, fully expecting to find this subject cleared up. But we were doomed to disappointment. We excused the author, however, from the fact that his book became large enough before he got to this important part of the subject. But, in course of time, the author became junior editor of the "American Journal," and then we hoped for light on the subject, for there he had *room to magnify his office*, as, according to a late contributor, they are "obliged to fill up three-fourths of their pages with 'selected articles.'" (I suppose Dr. V. referred to the American Journal, as the statement is true of no other one.) We would feel like complaining of the junior's long neglect of this matter were it not for the statement in his late editorial, "that no *sciolist* can grapple with this question." This is ample apology, and we must rest content.

With regard to the sub-phosphate of lime in defective dentition, we and others have used it a long time, have watched our cases closely, and are, thus far, satisfied with the results. We don't care if "the treatment is an old one for rickets," especially as we are not just now prescribing for rickets. When we become dissatisfied with the treatment we will abandon it.

We are gravely told, in this connection, that "there is something more to be considered than the mere question of plus and minus in animal chemistry," which statement is almost as interesting as the announcement of his belief in chemistry, and would probably be a very appropriate remark, if any one had disputed the fact.

The editor zealously objects to our views of caries, yet he does not attempt to tell us how the different varieties of the disease are produced, nor, as we can see, why he objects to our views of the action nitric, sulphuric, hydrochloric, and lactic acids on the teeth. He reminds us of a retired steam-doctor, a friend and neighbor to a patient of ours. The patient desired an article of food to which we objected. "I think sir," said the steam-doctor, you're wrong. A sick body always 'ort to have what he wants." "Why?" said we. "Oh, 'cause," said he. "Because why?" "Why—'*caze*," said he; and thus the matter dropped.

At the Cleveland Convention, in reply to a question as to the acids liable to be generated in the system "from the use of sugar *and saccharine substances generally*," we mentioned glucic acid as one of them. "This," says the editor, "surprised us not a little." Farther down he says of this acid, "Its properties are little known," and yet he speaks as positively in regard to it as though he knew them all by intuition.

Well, for some time before the Cleveland meeting, we were experimenting with "sugar and saccharine substances generally," with reference to the chemistry of caries, and at the meeting we spoke our candid sentiments. Experiments since have led us to doubt their correctness. But if we have made as many mistakes in all the dental meetings we ever attended as the editor here makes in a few sentences, we hope a generous profession will forgive us.

"The saccharine substances used in food," he says, "are all made of cane sugar." Are they? What about the granular sugar found in honey? And that contained in fruits? And what if it were true that "the saccharine substances used as food are all made of cane sugar?" Is the editor ignorant of the fact that before even the alcoholic fermentation takes place, the cane sugar must be converted into fruit sugar? And that, in preserving acid fruits, the cane sugar used is likewise transformed? (See Regnault's Chemistry, vol ii, pp. 474–5.)

The editor tells us farther, "Some comments were also made upon the difference between the caries produced by hydrochloric and sulphuric acid, and that caused by nitric acid. Here again we have gratuitous hypotheses, resting upon no basis of observation."

If the junior editor were omniscient, or even omnipresent, this language would neither be presumptuous nor impertinent, but as it is, we must be allowed to think that while he was busy with the chemistry of copper-mines, others could make at least a few observations on that of caries, without his being aware of it.

We do not know what he would acknowledge as a "basis of observation." Perhaps the "pickling of a few teeth in sour-drops," as described on page 157 of a work on chemistry, might serve as a *basis*.

We regard the subject of caries as *the important* chemical question before the profession; and our views, as laid down in the September number of the Register, in regard to the action of the acids there considered, are the result of what some consider long and laborious investigation; yet this investigation, being unlike the pickling above described, may have had "no basis of observation." We, by no means intended to intimate that the several varieties of caries can be produced by no acids but those we had under consideration; but that they *can* be produced by these, we do confidently maintain.

The editor tells us that nitric acid "is indeed occasionally formed, under peculiar circumstances, from the oxydation of nitrogenous matters." The presence of air and moisture is all that is peculiar in the circumstances necessary to its formation in "the oxydation of nitrogenous matters;" for if oxygen and nitrogen unite at all, nitric acid is their favorite combination.

Farther down we are told, "that, chemically speaking, no acid can act in any other way than dissolving out the calcareous salts." Well, great men are allowed to differ; and *sciolists* should be granted the same privilege, through courtesy. But every one knows that chloride of calcium, which dissolves in one-third of its weight of cold water, is more soluble than sulphate of lime, (plaster of Paris;) and these are the salts resulting from the decomposition of the carbonate of lime by hydrochloric and sulphuric acids. And again, the sub-phosphate is very soluble in hydrochloric, but is decomposed by sulphuric acid, especially if alcohol be present, when the same almost insoluble sulphate of lime is formed. Is there not a striking difference in the tendencies of these two acids to dissolve out the calcareous salts? But again, every student knows that a dilute solution of hydrochloric acid will dissolve out the calcareous, and leave the gelatinous portion of bones, while the nitric, equally diluted, will dissolve or destroy the latter as well as the former. In "white decay" the gelatinous portion of the tooth is disintegrated, or destroyed, while, in a different variety of the disease, it remains almost entire, in the cavity of decay. In the one case the animal portion is as really dissolved out as the earthy; in the other, it is not. The *junior* editor needs a little *practical* observation here.

But the junior editor has kindly told us that we are entirely mistaken in regard to the action of the acids which we have already considered, and has warned his readers against the serious errors which we have set forth. Well, rather than fail to appreciate such *disinterested* kindness, we will assume, for the time being, that we are mistaken. And now, will he tell us just what acids do produce dental caries, how they do it, from what source they are derived, and why they produce the several varieties observed in practice? When he does, we will be happy to lay his remarks before our readers. In the meantime we will go on with our investigations.

A word more, and we are done. The motives of the junior editor, in writing the article under consideration, have been already suspected. It is thought, by some, that jealousy was at the bottom of it. Now, this is unjust. He only did it "lest the cause of science (should) suffer, and "the truth decline in value;" and even that much he did "with reluctance," and because he couldn't help it; for he says, himself, "we *can not* allow them to pass without protest." W.

### "ORIGINAL"—WHAT IT MEANS.

Some time last winter we received a neat pamphlet, purporting to be "*A Paper upon Diseases of the Teeth*, read before the Cook Co. Medical Society at Chicago. By W. W. ALLPORT, D. D. S." We would have noticed it editorially at the time, (for it was well worthy of notice,) had we not intended, as soon as we had room, to publish it in full. Dr. A. requested us, in case we used the whole or a part of it, to credit the "North Western Medical and Surgical Journal." Well, in this number we could have found room for it; but here we find it among the "ORIGINAL COMMUNICATIONS" of the American Journal. Published the third time, yet still original! Whom shall we credit now? How nice to have an article that is always original, no matter where you find it! When they become common publishers "will not be obliged to fill up three-fourths of their pages with selected articles." How do you like the idea, brethren?

In the mean time, a dispute between Dr. A. and ourselves was settled, by the publication of his paper. He always told us he could not write, and we as constantly insisted that he could. When his pamphlet arrived we recorded a verdict in our own favor.

### DENTAL CONVENTION OF NORTHERN OHIO.

We recently attended the second meeting of this Convention. The brethren there are enlisted for the war. Their zeal and energy are really refreshing. There is possibly something in the lake breezes which imparts professional zeal. We are glad that a permanent association is about to be formed, as a result of these meetings. There is also a prospect of a local society in Cleveland and vicinity. When the members of the profession meet for mutual improvement, the cause of science must prosper. Northern Ohio is going—now, what about *Southern* Ohio, fellows?

### LOCAL ANÆSTHESIA.

We have in the past few days made some experiments in local anæsthesia; employing Dr. J. B. Branch's instrument for the purpose. The first case was that of a lady for whom we removed nine teeth, five anterior, and four posterior. In this case it was a success.

The application was continued in each case two and a half minutes. It was made to the four superior incisors, and they were all removed in a few seconds without any pain whatever, as the patient averred; afterwards the application was made to two molars on each side, and they were removed, quite as satisfactory to the patient as the others. Then the root of a large superior canine, deeply decayed, was removed, without pain. The patient was quite feeble and of nervous temperament, and was under considerable excitement before the operation was commenced.

The patient remarked, after the teeth were removed, that she could sit and have teeth extracted all day, in that way.

The gums soon after the operation regained the natural appearance, and four days afterward were in as good condition as they are ever found at that period.

In another case removed the root of a lateral incisor, it was very long, and on the point an abscess, and altogether in such a condition that it would have been exceedingly painful to remove in the ordinary manner.

Complete anæsthesia was produced in two and a half minutes, and the root removed without pain.

We have witnessed two or three other cases that were successful as the above.

Two cases were not successful; in one a very firmly set root of a second superior molar, very much decayed, the first and third molar both sound; the application was made without shielding the contiguous teeth, the cold produced pain in them.

The constitution was a vigorous, active one, and reaction very soon occurred. A protracted effort was made, for the removal of the root, in the mean time reaction took place, and great pain was produced. The contact of the preparation was not as perfect as could have been desired. By proper care in all the particulars, even a case of this kind could be met. In this as in many other things much depends upon details. Every thing must be just right, and properly applied to secure success. Promptness and dispatch is necessary in all the steps of the operation; one tardy movement may prove fatal to the success.

Dr. Branch has brought this local anæsthesia matter to great perfection. It, like many other things, requires skill and experience.

Dr. Branch is now visiting the members of the profession, with a view of giving them the advantage of his experience in regard to this subject, which has run through the past three or four years. Any one who thinks of using this method, should, if possible, avail himself of the Dr's. assistance. T.

---

### CLOSE OF VOLUME ELEVEN.

The present number closes volume eleven, and while we confess much delinquency and shortcoming, yet we hope something has been accomplished through the pages of the Register for the promotion of dental science.

For this the profession are indebted very much to the special friends of the Register, who have aided us by contributions from their pens, as well as sustained it with their money; and given us words of cheer and encouragement as we have passed along through the arduous way.

We design at the end of the present volume to remodel our mail list; in doing so one or two things will be accomplished. The names of those who have paid nothing on vols. x, and xi,—particularly those who have not felt interested enough to let us know whether they are in existence or not, will be dropped from the list.

Many of our subscribers, we are happy to say, are prompt in the payment of their dues; others are *not:* and we think two years is long enough to furnish them with a journal gratis. The majority of our subscribers communicate with us occasionally, and intimate to us their desires and intentions. To those who are in arrears for vols. x, and xi, we would say, you will understand the matter, if you do not receive the Register in future, at least till the old score is settled. We do not wish to be captious but it is a matter of self preservation.

If any who are still in arrears, and circumstances render them unable to pay at present, and they wish to continue the Register, they will please signify these facts to us, and we will be disposed to favor them a while longer. But those delinquents from whom we can not hear will be dropped at once.

We hope our friends will remember that we wish advance payment as far as convenient.

### GOLD FOIL.

Some time ago we received a specimen of Gold Foil, of Dr. A. M. Leslie, of St. Lous, manufactured of scraps of foil without re-melting.

It worked as well as any foil we have used, it was soft and very adhesive. With it some good fillings well made, as we think, with as much ease, as with any other make of foil.

We felt then as though we wanted " all of our foil made of scraps."

The appearance was similar to common foil, but it worked well and welded perfectly.

We have also received a specimen of No. 4 adhesive Gold Foil, manufactured by Morgan & Co., of Philadelphia, that possesses the adhesive property as fully as any foil we have ever seen. So it seems the art of making adhesive foil is no secret, the secret seems to have been on the other side. Foil makers we believe are able to give the profession almost anything they desire, in the form of foil. Formerly it was an object to avoid the adhesive property of gold, now it is desirable by most of our best operators.

### PRIZE ESSAY.

It is proposed in a short time, to issue another edition of the Prize Essay of the Mississippi Valley Association.

This is a popular treatise upon the teeth, their diseases and treatment, designed for gratuitous distribution by members of the profession to their patients; with a view of educating them properly in regard to the care of the teeth.

We have distributed two or three hundred copies in the last two years, and find the following results. Our patients have a higher appreciation of their teeth; and understand better the treatment they require, the great necessity of keeping them clean, and in a perfect condition. The teeth are attended to more promptly; decayed teeth receive attention at an earlier period, and consequently are more efficiently treated. The subject of dentition is much better understood. The object of the work is to give such information as the people require for the preservation of their teeth. Any members of the profession who may wish to procure a lot for distribution will please notify us of the fact immediately, as we wish to issue as soon as we have sufficient call to warrant us in doing so.

The committee on the Prize Essay are Drs. Richardson and Taft, either of whom may be addressed in regard to it.

---

### NEW DENTAL LATHE.

We notice at Toland's Depot, a portable foot lathe, got up by Jones, White & McCurdy, that we think is far better than any thing of the kind we have seen. It can be taken apart, and packed in a box 15 inches square. It can be taken down and put up in a few minutes; the height of the lathe can be changed. It runs very steady and is not liable to get out of repair. Brushes, stones and burrs can be adjusted to it; indeed, all the appliances that the dentist ever requires.

They are neat, and may be placed in the operating room. Jones, White and McCurdy say " it is just the thing," and we are disposed to believe them.

---

### THE DENTAL REPORTER.

The indefatigable J. T. Toland, ever on the alert for the good of the profession—has concluded that he is not doing enough for the profession, by selling dental goods, and so has assumed the editorial tripod. The first number of the Dental Reporter is the result. Who would have thought that there is so much editor in such a business man as John.

This is a neatly gotten up Journal, and contains much that is interesting to the profession.

We presume John's modesty accounts for the very low price which he sets upon his own productions. We hope he will recover.

